# Supplementary material for: Deep Eutectic Solvents as New Reaction Media to Produce Alkyl-Glycosides Using Alpha-Amylase from Thermotoga maritima
Source: Int J Mol Sci. 2019 Oct 31;20(21):5439. doi: 10.3390/ijms20215439 (PMC6862209; doi:10.3390/ijms20215439)
Supplement: Supplementary file 1 [file ijms-20-05439-s001.pdf]

Supplementary Information

Table S1. Deep eutectic solvents prepared in this work.

#	DES	Mol ratio[1]	Tm °C[1]	Method of preparation[1]
	**Amides**			
**1**	Choline chloride : urea	1 : 2	12	Heating under vaccum
**2**	Choline chloride : methyl urea	1 : 2	29	Heating under vaccum
	**Acids**			
**3**	Choline chloride : malonic acid	1 : 1	10	Heating
**4**	Choline chloride : levulinic acid	1 : 2	RT	Heating
	**Alcohols**			
**5**	Choline chloride : ethylene glycol	1 : 2	-10	Heating
**6**	Choline chloride : D-isosorbide	1 : 2	RT	Heating
**7**	Methyitriphenylphosphoniumbromid : glycerol	1 : 2	26	Heating
**8**	Choline chloride : glycerol	1 : 3 o 1 : 2	-20	Heating
**9**	Choline chloride : benzyl alcohol	1 : 2	RT	Heating
	**Natural DES (NADES)**			
**10**	Choline chloride : 1,3-propanediol : water	1 : 1 : 1	-110	Heating up to 50 ℃ (no vacuum)
**11**	Choline chloride : sucrose : H_2O	4 : 1 : 4	-83	Heating
**12**	Choline chloride : glucose : H_2O	5 : 2 : 5	-84	Heating

[1]The molar ratio is given as well as the melting point of the eutectic mixture if known. The solvents were prepared by the method indicated.

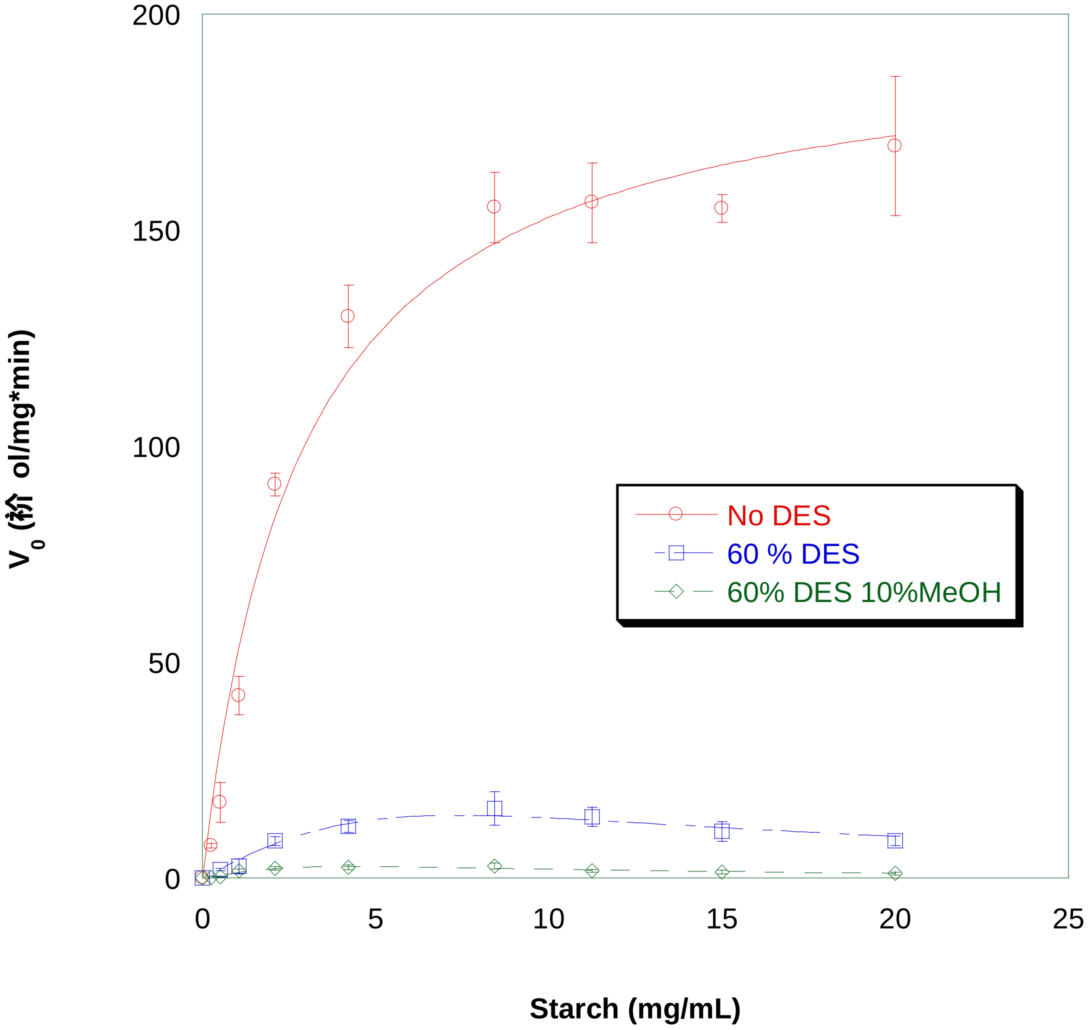

Figure S1. Non-linear curve fitting to Michaelis-Menten or inhibition product equations. Initial velocities (μmol/mg*min) were plotted against increasing concentrations of starch (mg/mL). In the case of the reactions carried out in DES, viscosity became a limiting factor at high starch concentrations. Error bars correspond to standard deviation of triplicates.

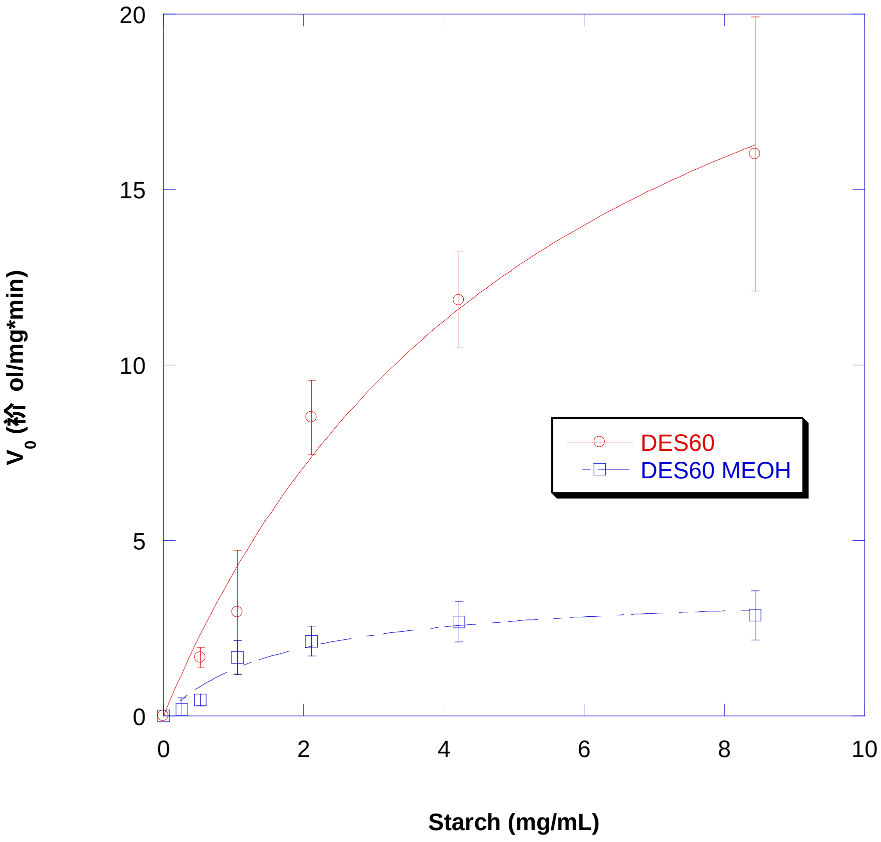

Figure S2. Initial velocities vs Starch concentration in DES and DES:MeOH medium. Since viscosity became a limiting factor, data at starch concentrations above 8 mg/mL were discarded, and the remaining data were fit to the Michaelis-Menten equation. Error bars correspond to standard deviation of triplicates.

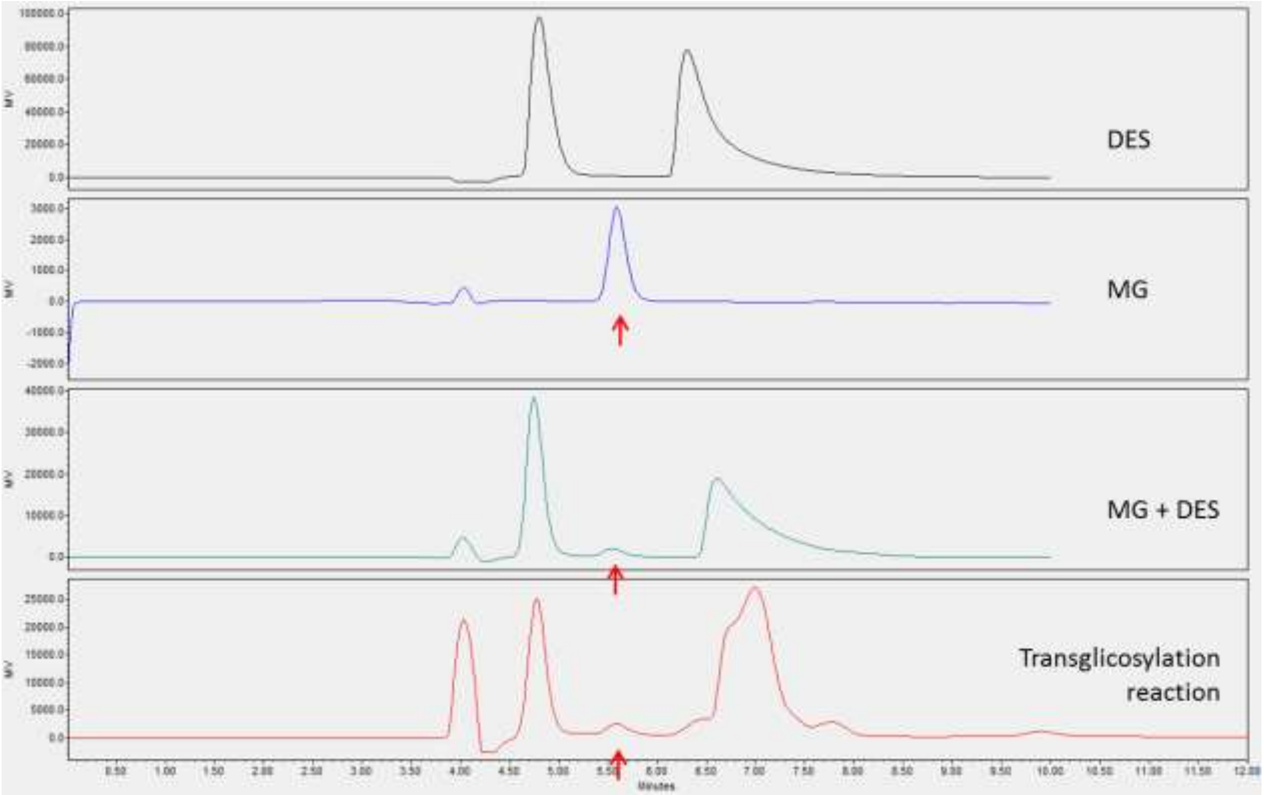

Figure S3. Chromatographic profile of standards and alcoholysis reaction products. In black, 80% DES; in blue, methyl glucoside (MG) directly dissolved in mobile phase (ACN:H20, 80:20); in dark green, MG dissolved in 40% DES; in red, alcoholysis reaction products (includes a mixture of dextrins, glucose, methanol, DES and MG). Samples from reactions were previously diluted 1:1 with buffer and digested for 6 h with glucoamilase from *A. niger* to hydrolyze remaining amylose. After digestion, samples were filter through a 0.22 μm nylon membrane and injected into a Gold-amino column. The red arrow shows the peak corresponding to the product of interest MG to be quantified.
